# Characterization of Flavonoids and Naphthopyranones in Methanol Extracts of *Paepalanthus chiquitensis *Herzog by HPLC-ESI-IT-MS^n^ and Their Mutagenic Activity 

**DOI:** 10.3390/molecules18010244

**Published:** 2012-12-27

**Authors:** Fabiana Volpe Zanutto, Paula Karina Boldrin, Eliana Aparecida Varanda, Samara Fernandes de Souza, Paulo Takeo Sano, Wagner Vilegas, Lourdes Campaner dos Santos

**Affiliations:** 1 Department of Organic Chemistry, Institute of Chemistry, UNESP — Sao Paulo State University, Araraquara CEP 14800-900, Sao Paulo, Brazil; 2 Department of Biological Sciences, Faculty of Pharmaceutical Sciences of Araraquara, UNESP — Sao Paulo State University, Araraquara CEP 14801-902, Sao Paulo, Brazil; 3 Institute of Biosciences, Sao Paulo University, Sao Paulo CEP 05508-900, Sao Paulo, Brazil; 4 Experimental Campus of Sao Vicente, UNESP — Sao Paulo State University, Sao Vicente CEP 11350-000, Sao Paulo, Brazil

**Keywords:** *Paepalanthus*, naphthopyranones, flavonoids, mutagenicity

## Abstract

A HPLC-ESI-IT-MS^n^ method, based on high-performance liquid chromatography coupled to electrospray negative ionization multistage ion trap mass spectrometry, was developed for rapid identification of 24 flavonoid and naphthopyranone compounds. The methanol extracts of the capitulae and scapes of *P. chiquitensis *exhibited mutagenic activity in the *Salmonella*/microsome assay, against strain TA97a.

## 1. Introduction

Eriocaulaceae is a pantropical, predominantly herbaceous monocotyledonous family, comprising around 1,200 species in 10 genera [1]. They are common and diagnostic components of the herbaceous rocky outcrop vegetation of Brazil called “campos rupestres”, which flourishes at elevations exceeding 900 m above sea level. *Paepalanthus *is the largest genus in this family, with approximately 500 species, more than 400 occurring only in Brazil [2].

Taxonomic studies to delimit the genus, whose definition remains controversial, and the biological investigation of molecules isolated from Eriocaulaceae are of great importance, especially because several molecules possess antioxidant [3,4], cytotoxic and mutagenic activities [5,6,7] and some extracts of the assayed plants show antiulcerogenic activity [8].

Flavonoids have frequently been used in chemotaxonomy, because they are widespread, their patterns tend to be specific, they are relatively stable and their biosynthesis/accumulation is largely independent of environmental influence [9]. In our laboratories, glycosylated acyl flavonoids and quercetin derivatives with one sugar unit have been isolated from *Paepalanthus *genus [2,10,11,12,13].

Naphthopyranones are a class of natural metabolites, described until now only in the capitulae of the *Paepalanthus *genus, displaying anti-inflammatory [14] and cytotoxic activities [15]. Naphthopyranone derivatives are found in all the *Paepalanthus* species belonging to the subgenus *Platycaulon * [2,7,10,11,16]. However, there are no chemical and biological data for *Paepalanthus chiquitensis* Herzog (formally cited as *Paepalanthus giganteus* Sano), section *Diphyomene * [17].

Plants are valuable sources of potential chemotherapeutic drugs and are used to treat many ailments, but some medicinal plants and their compounds can be dangerous to human health [18,19,20]. The *Salmonella* mutagenicity test (Ames test) detects if any sample provokes specific mutations of the genetically modified DNA of selected *S. typhimurium *strains and is used worldwide as an initial screening of the mutagenic potential of new chemicals for hazard identification and for the registration or acceptance of new chemicals by regulatory agencies and an important component for making the bacterial mutagenicity test useful was the inclusion of an exogenous mammalian metabolic activation system, because bacteria are unable to metabolize chemicals via cytocromes P450, as in mammals and other vertebrates. Many carcinogens remain inactive until they are enzymatically transformed to an electrophilic species that is capable of covalently binding to DNA, leading to mutation [21]. 

In the present paper, methanol extracts of the capitulae and scapes of *P. chiquitensis* was prepared and analyzed by HPLC-ESI-ITMS^n^ and assayed by the Ames test in the * S. typhimurium *tester strains TA98 and TA97a (to detect frameshift mutations), TA100 (detects base-pair-substitution mutations) and TA102 (normally used to detect mutagens that cause oxidative damage and base-pair-substitution mutations) in presence and absence of metabolic activation system and the results compared with those from other *Paepalanthus *species.

## 2. Results and Discussion

**Figure 1:**
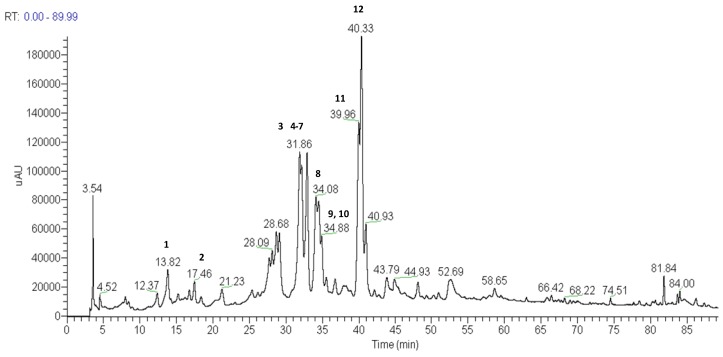
Total ion current chromatogram of the methanol extract of capitulae of *P. chiquitensis* (HPLC-ESI-IT-MS^n^ negative ion mode). For conditions, see experimental part.

**Figure 2:**
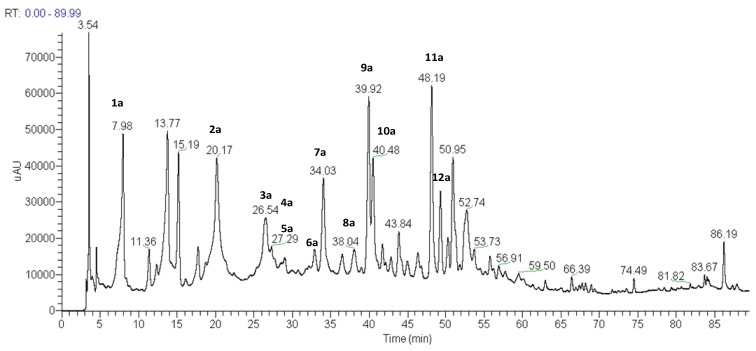
Total ion current chromatogram of the methanol extract of scapes of *P. chiquitensis* (HPLC-ESI-IT-MS^n^ negative ion mode). For conditions, see experimental part.

In this study, methanol extracts of the capitulae and scapes of *P. chiquitensis* were prepared and analyzed by HPLC-ESI-ITMS^n^. Several experiments were performed to establish suitable HPLC conditions. The best results were obtained with a Phenomenex Synergi Hydro RP-80 C_18_ column eluted with a water/methanol gradient acidified with acetic acid, as described in the experimental section. The total ion current chromatograms of the two extracts generated by negative ion HPLC-ESI-IT-MS^n^ analysis are shown in Figure 1 and Figure 2. The UV spectra were also recorded, since they provide useful data for the identification of different compounds exhibiting particular UV absorbances.

The HPLC-ESI-MS^n^ analyses of compounds present in the methanol extracts of capitulae and scapes of *P. chiquitensis* led to the detection of flavonol and flavanonol derivatives and the presence of naphthopyranone derivatives (Table 1 and Table 2 and Figure 1 and Figure 2). 

**Table 1:** ESI-MS and ESI-MS^n^ product ions of compounds occurring in the methanol extracts of capitulae *from P. chiquitensis*.

Substance	Peak	T_R_	UV spectra λmax (nm)	[M−H]^−^	Major MS^2^ and MS^3^ fragments
6-Methoxyquercetin-7-*O*-*β*-D-glucopyranosyl-(6→1)-*O*-*β*-D-glucopyranoside	1	13.82	235, 345	655	640,493, 331, 316
6,3'-Dimethoxyquercetin-7-*O*-*β*-D-glucopyranosyl-(6→1)-*O*-*β*-D-glucopyranoside	2	17.46	235, 345	669	507, 345, 330, 302, 287
7-Methoxyquercetin-*O*-hexose	3	31.86	268, 342	477	315, 301, 273
6-Hydroxy-7,3,4-trimethoxyflavanonol-di-*O*-hexose	4	32.14	259, 349	685	623, 315
6-Methoxykaempferol-3-*O*-*β*-D-6-(*p*-coumaroyl)glucopyranoside	5	32.14	270, 315	623	608, 477, 300
6-Hydroxy-7-4-dimethoxyquercetin-3-*O*-hexose	6	32.31	257, 350	507	477, 315
6,3-Dimethoxyquercetin-3-*O*-*β*-D-6-(*p*-coumaroyl)glucopyranoside	7	32.88	262, 358	653	345, 330, 287
5-10-Ddihydroxy-7-methoxy-3-methyl-1*H*-naphtho[2,3c]pyran-1-one-9-*O-α-*L-rhamnopyranosyl-(1→6)- *O*-*β*-D-glucopyranoside	8	34.08	271, 280sh, 383	595	449, 287
10-Hydroxy-5,7-dimethoxy-3-methyl-1*H*-naphtho[2,3c]pyran-1-one-9-*O*-*β*-D-allopyranosyl (1→6)-*O-β*-D-glucopyranoside	9	34.48	273, 283sh, 362	625	593, 463, 301
Quercetin-3-*O*-di-hexose	10	34.88	252 281sh, 362	625	609, 447, 285
10-Hydroxy-7-methoxy-3-methyl-1*H*-naphtho[2,3c]pyran-1-one-9-*O*-*β*-D-glucopyranoside	11	39.96	272,280sh381	433	271, 256
10-Hydroxy-5-7-dimethoxy-3-methyl-1*H*-naphtho[2,3c]pyran-1-one-9-*O*-*β*-D-glucopyranoside	12	40.33	273,284sh, 384	463	301, 286, 272, 256

**Table 2:** ESI-MS and ESI-MS^n^ product ions of compounds occurring in the methanol extracts of scapes from *P. chiquitensis*.

Substance	Peak	T_R_	UV spectra λmax (nm)	[M−H]^−^	Major MS^2^ and MS^3^ fragments
6-Hydroxyquercetin-3-*O*-di-hexose	1a	7.98	260,295sh, 340	641	479, 317
6-Hydroxyquercetin-3-*O*-hexose dimer	2a	20.17	260,274sh, 348	958	479, 463
6-Metoxykaempferol-3-*O-*hexose-*O*-pentose	3a	26.54	267, 337	577	431, 299
4-Methoxyapigenin-7-*O*-(3-galloyl)-*α*-D-arabinopyranosyl-(2→1)-apiofuranosyl-(3→1)- *α*-D-arabinopyranoside	4a	26.94	267,337	831	803, 635, 623, 605, 315, 269
6-Methoxyquercetin-7-*O*-glucoside	5a	27.29	253sh, 345	493	331, 316
6,3-Dimethoxyquercetin-3-*O*-*β*-D-6-(*p*-coumaroyl)- glucopyranoside	6a	32.89	262sh,358	653	345, 330, 287
5-10-Dihydroxy-7-methoxy-3-methyl-1*H*-naphtho[2,3c]pyran-1-one-9-*O-α*-L-rhamnopyranosyl-(1→6)- *O*-*β*-D-glucopyranosíde	7a	34.03	268,279sh,349	595	449, 287
Flavanonol-di-*O*-hexose	8a	38.04	242,279sh,325	627	465, 303
10-hydroxy-7-methoxy-3-methyl-1*H*-naphtho[2,3c]pyran-1-one-9-*O*-*β*-D-glucopyranoside	9a	39.92	270,279sh, 387	433	271, 256
6-Hydroxy-7-methoxyquercetin-3-*O*-pentose	10a	40.48	250,283sh, 326	463	433, 331
6-Hydroxy-7,3,4-trimethoxyflavanonol	11a	48.19	242,261sh, 324	361	346, 331, 316
6-Hydroxy-7,4-dimethoxyquercetin-3-*O*-hexose	12a	49.31	250,283sh, 326	507	345, 286

The full negative ESI-MS spectrum of compound **4a** showed an [M−H]^−^ ion at *m/z* 831. ESIMS^2^ spectrum highlighted the presence of the aglycone ion at *m/z* 269, due to simultaneous loss of three sugar units and a galloyl unit.

The ^1^H-NMR spectrum of compound **4a **exhibited signals for the aromatic protons at δ 7.96 (2H, *d*, *J* = 8.0 Hz, H2'/6'), 6.95 (2H, *d*, *J* = 8.0 Hz, H3/5), 6.79 (*s*, H-8), which revealed the substitution patterns in the B ring, while those at δ 6.42 (1H, *d*, *J *= 2.0 Hz) and 6.85 (1H, *d*, *J* = 2.0 Hz) corresponded to H-6 and H-8, respectively in the A ring. There was also a signal for hydrogen at δ 6.79 (2H, *s*). This signal showed correlations in the *g*HMBC contour map with carbons at δ 112.5, 147.0, 121.2, 149.6 and 165.0, confirming the presence of a galloyl unit. The location of a methoxy group was also confirmed by the *g*HMBC experiment, since the signal at δ 3.74 (3H, *s*) correlated with the carbon at δ 161.0 (C4).

Analysis of the ^1^H-NMR spectrum in the region of the sugars showed signals of anomeric protons at δ 5.11 (1H, *d*, *J* = 8.0 Hz), 5.34 (1H, *d*, *J* = 1.0 Hz) and 5.16 (1H, *d*, *J* = 7.0 Hz). Analysis of the signals in the TOCSY experiment suggests two possible arabinose units with α configuration. The chemical shift (deshielded) on the anomeric carbon at δ 108.0 suggested that one of the units of sugars could be apiose.

In the TOCSY experiment, we confirmed the spin systems of each sugar unit, because the signal radiated at δ 5.11 showed consistency with the transfer of signals at δ 3.82, 3.70, 3.64 and 3.38. Irradiation of the hydrogen in the signal at δ 5.34 showed correlation with the signals of protons at δ 3.74 and 3.38 and, finally, irradiation of the hydrogen signal at δ 5.16 correlated with the signals of protons at δ 3.50, 3.46, 3.38 and 3.20.

**Figure 3:**
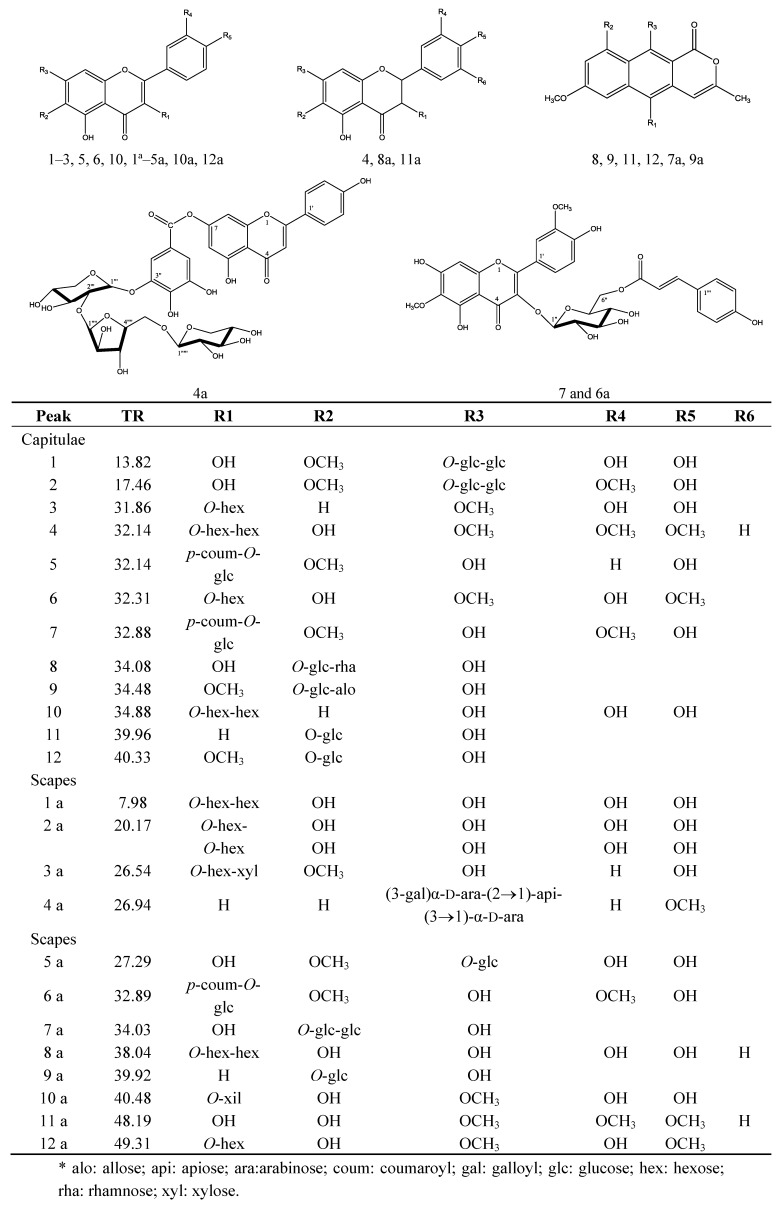
Structure of the compounds identified in the capitulae and scapes from *P. chiquitensis.*

The sequence of the hydrogens in each system was confirmed in the COSY experiment. The correlations of the *g*HSQC experiment enabled the respective carbons to be assigned (Table 3). The deshielded C-7 (δ164, 0) suggests that the carbon is replaced. In the *g*HMBC spectrum, correlations were observed between arabinose anomeric hydrogen at δ 5.11 and the carbon of the galloyl C-3" (δ 149.6). This experiment also showed that the hydroxyl at C2 (δ 72.8) of arabinose was replaced. This inference was made by observing the correlation of the apiose hydrogen signal at δ 5.34 with carbon C2 (δ 72.8) of the arabinose. The experiment shows *g*HMBC correlation of anomeric hydrogen δ 5.16 of the arabinose with the carbon of apiose at δ 79.0. Consequently, **4a** was determined to be the new 4-methoxyapigenin-7-*O*-(3-galloyl)-*α*-D-arabinopyranosyl-(2→1)-apiofuranosyl-(3→1)-*α*-D-arabinopyranoside (Figure 3, Table 3).

**Table 3:** ^13^C and ^1^H-NMR Data (*J* in Hz) for the compounds **4a**, **7** and **6a** (500 MHz, δ ppm, in DMSO-*_d6_*).

4a			7 and 6a	
Position	δ_H_ (*J* in Hz)	δ_C_	δ_H_ (*J* in Hz)	δ_C_
2		164.1	-	156.4
3	6.85 s	103.9	-	132.6
4	-	180.0	-	177.0
5	-	161.8	-	152.5
6	6.42*d* (2.0)	99.0	-	131.6
7		162.6	-	157.4
8	6.85 *d* (2.0)	95.0	6.49 *s*	94.6
9	-	157.0	-	152.2
10	-	105.0	-	104.8
1	-	121.0	-	121.1
2	7.96 *d* (8.0)	129.3	7.55 *dd* (8.5, 2.0)	116.4
3	6.95 *d* (8.0)	116.5	6.85*d* (9.0)	147.0
4	-	161.0	-	149.0
5	6.95 *d* (8.0)	116.5	6.76 *d *(8.5)	115.2
6	7.96 *d* (8.0)	129.3	7.51 *dd* (8.5, 2.0)	121.6
OCH_3_-4	3.74 *s*	56.0	-	-
OCH_3_-3	-	-	3.82	56.7
OCH_3_-6	-	-	3.70	60.0
	galloyl		glucose	-
1		121.2	5.43 *d* (7.5)	100.8
2	6.79 *s*	112.0	3.37 *dd* (9.0; 7.5)	73.4
3	-	149.6	3.48 *dd* (9.0; 9.0)	76.8
4	-	139.0	3.27 *dd *(9.0; 9.0)	69.8
5	-	-	3.29 *m *	75,6
6	6.79 *s*	-	4.24 *dd* (6.0; 10.0)4.02*dd* (5.0; 12.0)	62.8
α	-	147.0	-	-
β	-	112.5	-	-
(C=O)	-	165.0	-	165.3
	arabinopyranosyl		coumaroyl	
1	5.11 *d* (8.0)	98.0	-	125.0
2	3.82 *dd* (5.5; 8.5)	72.8	7.36 *d* (8.5)	130.0
3	3.70 *dd* (3.5, 7.5)	76.7	6.78 *d* (8.5)	116.2
4	3.64 *m*	74,6	-	159.4
5	3.38 *dd* (11.5)3.60 *d* (11.5)	60.5	6.78 *d* (8.5)	116.2
6′	-	-	7.36 *d* (8.5)	130.0
α	-	-	6.14 *d* (16.0)	114.5
β	-	-	7.31 *d* (16.0)	144.4
	apiofuranosyl			
1	5.34 *d* (1.0)	108.0	-	--
2	3.74 *d (3.0)*	76.7	-	-
3	-	79.0	-	-
4	3.64 *d (5.5)*	70.4	-	-
5	3.32 *dd *	64.9	-	-
	arabinopyranoside			-
1	5.16*d* (7.0)	97.0	-	-
2	3.46 *dd (5.5, 7.5)*	72.8	-	-
3	3.50 *dd* (3.5; 7.5)	69.4	-	-
4	3.20 *m*	71.6	-	-
5	3.38 *d *(11.5)3.62 d (11.5)	62.5	-	-

The compounds **8**, **9**, **11**, **12** from the capitulae and **7a** and **9a** from the scapes of *P. chiquitensis* are in a class of substances, common in *Paepalanthus,* known as naphthopyranones. These were detected in greater quantities in the capitulae at the retention times: 34.08, 34.48, 39.96 and 40.33 min and in the scapes at 34.03 and 39.92 min.

These compounds were identified by comparing the peaks of their UV spectra with those of the available references in the literature. The naphthopyranones have characteristic bands at 270–273 and 280–284 nm [10,11,13,22,23].

The substances **11** and **9a** (λ_max_ = 272, 280, 381 nm) show a molecular ion at *m/z *433, suggesting a paepalantine derivative [M−H]^−^ [18]. The second-generation ion product spectrum of precursor ion at *m/z* 433 shows the loss of a hexose at *m/z* 271 [M−H−162]^−^. The MS^3^ showed a peak at *m/z* 256 that refers to the loss of one hexose and a methyl group [M−H−162−15]^−^. The information in the UV spectrum and the characteristic fragmentation of naphthopyranones lead us to suggest that this substance is 10-hydroxy-7-methoxy-3-methyl-1-H-naphtho[2,3c]pyran-1-one-9-*O-β*-D-glucopyranoside [22,23,24].

The substances **8 **and **7a** (λ_max_ = 270, 279, 346 nm) show the molecular ion at *m/z *595 [M−H]^−^. The second-generation ion product of this ion at *m/z *449 suggests the loss of rhamnose [M−H−146]^−^ and the fragment ion at *m/z* 287 suggests the loss of two hexoses [M−H−146−162]^−^. On the basis of these fragmentation patterns in the MS/MS and UV experiments, we suggest the presence of 5-10-dihydroxy-7-methoxy-3-methyl-1*H*-naphtho[2,3c]pyran-1-one-9-*O-α*-L-rhamnopyranosyl-(1→6)-*O*-*β-*D-glucopyranoside) [22,23].

The other paepalantine derivative common in *Paepalanthus* species is paepalantine-9-*O*-*β*-D-allopyranosyl-(1→6)-*O*-*β*-D-glucopyranoside, (**9**) [23,24]. This substance was identified at the retention time of 34.48 min, with a UV spectrum characteristic of paepalantine (273, 283, 362nm). The deprotonated molecular ion was identified at *m/z *625 [M−H]^−^. The MS^n^ spectrum shows characteristic fragmentation at *m/z* 593 [M−H−31]^−^, 463 [M−H−162]^−^ and 301 [M−H−(2 × 162)]^−^, respectively, suggesting the loss of two hexoses and a methoxy group. This substance was detected only in the capitulae of *P. chiquitensis.*

Finally, in the capitulae (T_R_ = 40.33 min), we detected another paepalantine derivative (**12**) with UV spectrum (λ_max_ = 273, 284, 384 nm). The ESI-MS spectrum showed the deprotonated molecule at *m/z* 463 [M−H]^−^. The MS^n^ spectrum showed the characteristic fragmentation sequence at *m/z* 301 [M−H−162]^−^, 286 [M−H−15−162]^−^, 272 [M−H−162−(2 × 15)]^−^ and 256 [M−H−162−(3 × 15)]^−^. These data suggested that this molecule is 10-hydroxy-5-7-dimethoxy-3-methyl-1H-naphtho[2,3c]pyran-1-one-9-*O*-*β*-D-glucopyranoside, a naphthopyranone previously isolated from *P. bromeliodes*, *P. hilairei *and *P. ramosus* [10,22,23,25].

Compounds **4** of the capitulae and **8a**, **11a** of the scapes, with retention times 32.14 and 38.04 and 48.19 min, produce UV spectra characteristic of flavanonol [9,25]. The ESI-MS of the compound **4** shows the deprotonated molecular ion at *m/z* 685, suggesting that it is a flavanonol with the molecular formula C_30_H_38_O_18_. The ion at *m/z* 623 refers to the loss of two methoxyl groups and the fragment ion at *m/z* 315 refers to the loss of two methoxyl groups and two hexoses.

The ions produced from the scape extract showed fragments characteristic of the diglycoside flavanonol in **8a** at *m/z* 627 [M−H]^−^. This is evidence of the loss of hexose at *m/z* 465 [M−H−162]^−^ and two hoxoses at *m/z* 303 [M−H−(2 × 162)]^−^.

Finally, at *m/z* 361 in **11a**, we suggest the 6-hydroxylated flavanonol with three methoxyl groups. These flavanonols are not common in the Eriocaulaceae, but these is a report of the isolation, in methanolic extract of the leaves of *Paepalanthus argenteus* var. *argenteus* (Bongard) Hensold, of a flavanonol characterized as xeractinol. This dihydroflavonol served as a taxonomic marker of *Paepalanthus* subg. *xeractis* [26]. Since such flavanonols have been identified in *P. chiquitensis*, we suggest that this class of compounds can be of use in the chemotaxonomy of *Paepalanthus*.

Compound **2a**, with retention time 20.17 min in scape extract, shows a deprotonated molecule at *m/z *958 suggesting that it is a flavonoid dimer. The MS/MS spectrum shows a fragment at *m/z *479, indicating the fragmentation of a part of the dimer [M−H−C_21_H_19_O_12_]^−^, and another at *m/z* 463, indicating another part of the dimer [M−H−C_21_H_19_O_13_]^−^. This suggests that this molecule has the structure of a 6-hydroxyquercetin derivative, with a hexose on each part of the dimer. The connection between the two parts of the dimer could not be established by ESI-MS, requiring isolation and structural elucidation by NMR.

A kaempferol derivative **3a** containing a pentose was also detected at retention time 26.54 (in the scapes). The evidence for this is as follows: the deprotonated molecule was identified at *m/z* 577. The MS/MS spectrum showed the loss of rhamnose at 146 Da and MS^3^ showed the loss of a hexose and a pentose at *m/z *299 [M−H−146−132]^−^.

The signal at *m/z* 669 (**2**) also suggests a quercetin derivative [24]. The sequence of fragmentation in the MS^n^ spectra showed a hydroxydimethoxyquercetin with two hexoses. Specificaly, the fragmentation pattern exhibited by compound **2** was coherent with the 6-3-dimethoxyquercetin core supporting two hexosyl moieties, while the MS/MS experiment on compound **2 **showed a product ion at *m/z *345, due to the simultaneous elimination of two sugar units [M−H−162−162]^−^, and an ion at *m/z *330, due to the loss of a methyl group from the 6-methoxyquercetin core. NMR data of the isolated compound **2 **confirmed that it was 6-3-dimethoxyquercetin-3-*O*-*β*-D-glucopyranosyl-(6→1)-*O-β*-D-glucopyranoside [27]. 

A methoxyquercetin derivative was detected at the retention time (t_R_) 13.82 min in the capitulae extract. The ESI-MS spectrum showed the molecular ion (**1**) at *m/z* 655, the MS^2^ spectrum had a peak at *m/z* 493 [M−H−162]^−^ and MS^3^ had a peak at *m/z* 331 [M−H−162−162]^−^ showing the consecutive loss of two hexoses [24].

The ion with retention time 7.98 min (**1a**, scapes), showed a product ion at *m/z* 641. The MS^2^ spectrum shows a fragment ion at *m/z* 479 [M−162−H]^−^, due to the flavonol aglycone, originating by the loss of a hexose unit from the precursor ion, and an at ion a *m/z *317, due to the consecutive loss of two hexoses from the 6-hydroxyquercetin core [M−162−162−H]^−^.

A methoxyquercetin derivative was detected at the retention time of 27.29 (**5a**) min in the total ion current (TIC) profile of the scapes. The relative mass spectrum exhibited a peak at *m/z* 493 and the MS^2^ spectrum exhibited a fragment at *m/z* 331, due to the flavonoid aglycone, formed by the loss of a hexose unit from the precursor ion. We suggest that **5a** is the 6-methoxyquercetin-7-*O*-glucoside [28]. 

Another methoxyquercetin derivative was detected only at the retention time 31.86 min (**3**) in the extract of capitulae. The full negative ESI-MS spectrum showed an ion at *m/z* 477 [M−H]^−^ (Table 1). The MS^2^ spectrum showed a peak at *m/z* 315 [M−H−162]^−^, suggesting the loss of a hexose.

The flavonoid acyl glycosides are common in *Paepalanthus* species. These compounds were detected in *P. Chiquitensis *at retention times 32.14 and 32.88 min, in methanolic extracts from capitulae and scapes respectively (**5**, **7** and**6a**). 

The ESI-MS spectrum showed the ion of compounds **7** and **6a **at *m/z* 653 [M−H]^−^. The ESI-MS^2^ spectrum of this ion showed a representative ion at *m/z* 345, attributed to the loss of two hexoses [M−146−162−H]^−^, and the ion at *m/z* 330 showed the loss of the methyl group [M−H−146−162−15]^−^. The MS^2^ of the ion precursor at *m/z* 653 afforded an ion product at *m/z* 287, attributed to the loss of the other methyl group and CO [M−H−146−162−(2 × 15)−28]^−^.

The ^1^H-NMR spectrum of compound **7** and **6a** showed the proton signals that clearly indicated an OH group in a singlet at δ 12.71, due to hydrogen bonding to the C4 carbonyl. A doublet at δ 7.55 (*J* = 2.0 Hz), a double doublet at δ 7.51 (*J* = 8.5 Hz; 2.0 Hz) and a doublet at δ 6.76 (*J* = 8.5 Hz) are related to the B-ring of the aglycone moiety. The singlet at δ 6.47 was assigned to H8 of the A ring. These data, along with those derived from HSQC and HMBC experiments, allowed the aglycone moiety of **7** and **6a** to be identified as 6-methoxyquercetin. Two other doublets at δ 6.78 (*J* = 8.0 Hz) and δ 7.36 (*J* = 8.0 Hz) were attributed to H3/H5 and H2/H6 of the p-coumaroyl moiety, respectively. The two doublets (*J *= 16.0 Hz) at δ 6.15 and 7.33 were assigned to Ha and Hb of the *p*-coumaroyl moiety with *trans *stereochemistry, respectively. The signal at δ 5.43 (*J* = 7.5 Hz) was assigned to a D-glucose in the β-configuration. The singlets at δ 3.69 and 3.82 (3H each) indicated the presence of the two methoxy groups. The assignments of each signal, based on 2-D ^1^H-^1^H COSY, ^13^C-^1^H COSY and *g*HMBC spectra, are shown in Table 3. 

The signal at δ 63.1 (CH2) shows that the *p*-coumaroyl linkage was at C-6 of the glucose unit. The deshielding of C2, compared to patuletin (in which C2 is observed at d 147.1), indicated that position 3 should be substituted by the *p*-coumaroyl glucose moiety [29]. 

This evidence was confirmed by HSQC, HMBC, and COSY correlations. The downfield shifts of H-_6a-6b_ and C-_6_ of the glucose unit (δH 4.31 and 4.26; δC 62.8) suggested that the *p*-coumaroyl moiety was located at C-6glc. HMBC correlation between the two proton signals at δ 4.31 and 4.26 and the carboxylic carbon at δ 166.2 confirmed this assumption.

Thus, compounds **7** and **6a** are the new 6,3**-**dimethoxyquercetin-3-*O-β*-D-6-(*p*-coumaroyl)glucopyranoside. 

Another acyl glycoside identified was 6-methoxykaempferol-3-*O*-*β*-D-6-(*E*-*p*-coumaroyl)-glucopyranosíde (**5**) [23,30]. The deprotonated molecule was detected at *m/z* 623. The MS^2^ spectrum shows the loss of *p*-coumaroyl at *m/z* 477 [M−H−146]^−^. MS^3^ shows the fragment ion at *m/z* 300 that was attributed to the loss of *p*-coumaroyl and the one hexose and one methyl group [M−H−146−162−15]^−^ and finally at *m/z* 300, we detected the aglycone [M−H]^−^ which proved to be a derivative of kaempferol.

It can be seen therefore that most of the compounds in the methanolic extracts of capitulae and scapes of *P. chiquitensis* were basically flavonoids (quercetin derivatives) and naphthopyranones (paepalantine derivatives), illustrated in Figure 3. The contents of quercetin and paepalantine derivatives were determined in µg/100 mg of capitulae extract (335 ± 2.4 and 455 ± 3.3) and scapes (391 ± 1.1 and 431 ± 1.4) respectively [31].

Table 4 shows the mean number of revertants/plate (M), the standard deviation (SD) and the mutagenic index (MI) after treatments with the methanolic extracts of capitulae and scapes from *P. chiquitensis*, observed in *S. typhimurium* strains TA98, TA100, TA97a and TA102, in the presence (+S9) and absence (−S9) of metabolic activation. 

The *Salmonella *strains used in the test have different mutations in various genes in the histidine operon; each of these mutations is designed to be responsive to mutagens that act via different mechanisms [21]. A series of doses were used, from 0.6 to 11 mg/plate, and mutagenic activity was observed only with TA97a, both in the presence and absence of metabolic activation. These results reveal that the MeOH extracts from the capitulate and scapes of *P. chiquitensis* contains compounds that cause frameshift mutations by acting directly and indirectly on the DNA. Results were negative with strains TA100, TA98 and TA102, with or without S9.

Other studies on the methanolic extract of capitulae of Eriocaulaceae species have been carried out [5,6,7,32]. In these studies, the mutagenic activity was induced by naphthopyranones present in these parts. Mutagenicity studies carried out with naphthopyranones and flavonoids [7], led to the conclusion that the mutagenicity observed in strain TA97a for the methanol extracts of capitulae and scapes, was due to the naphthopyranone and quercetin derivatives present. The values of MI were higher for capitulae than for scapes and more naphthopyranone than quercetin derivatives were detected in the extract of capitulae. The naphthopyranone has hydroxyls at positions 1, 9 and 10, which are free to make hydrogen bonds with the DNA bases. In flavonoids, this mutagenic activity is due to a free hydroxyl at position 3, a double bond between positions 2 and 3, and a keto group at position 4, allowing the free hydroxyl in position 3 to tautomerise the molecule to a 3-keto molecule [20,33].

**Table 4:** Mutagenic activity expressed as the mean and standard deviation of the number of revertants/plate and the mutagenic index (MI), in bacterial strains TA98, TA97a, TA100 and TA102 treated with methanolic extract of capitulae and scapes of *P. chiquitensis *at various doses, with (+S9) or without (−S9) metabolic activation.

Treatments mg/plate	Number of revertants/plate in *S. typhimurium* strains (M ± SD) and (MI)							
	*TA98 TA97a TA100 TA102*							
MeOH Ext. Capitulae	−S9 ^b^	+S9 ^d^	−S9 ^b^	+S9 ^d^	−S9 ^a^	+S9 ^d^	−S9 ^c^	+S9 ^e^
0	24 ± 1	31 ± 2	133 ± 17	161 ± 24	185 ± 11	123 ± 9	382 ± 30	248 ± 3
0.62	--	--	--	--	--	--	245 ± 24 (0.6)	388 ± 71 (1.5)
1.25	--	--	--	--	--	--	277 ± 21 (0.7)	332 ± 64 (1.3)
1.87	34 ± 9 (1.8)	32 ± 1 (1.5)	753 ± 127 **(5.6)	1410 ± 172 **(8.7)	334 ± 21 (1.7)	193 ± 12 (1.6)	--	--
2.50	--	--	--	--	--	--	359 ± 53 (0.9)	350 ± 7 (1.4)
3.75	35 ± 8 (1.1)	29 ± 5 (1.3)	419 ± 108 *(3.1)	1358 ± 76 **(17.9)	187 ± 12 (0.9)	148 ± 7 (1.2)	421 ± 133 1.1)	341 ± 104(1.4)
7.50	57 ± 11 (1.8)	33 ± 4 (1.5)	698 ± 259 *(5.2)	1493 ± 62 **(24.0)	151 ± 24 (0.7)	172 ± 11 (1.4)	--	--
11.25	47 ± 8 (1.4)	39 ± 2 (1.8)	231 ± 68 (1.7)	1348 ± 127 **(10.5)	118 ± 13 (0.6)	156 ± 12 (1.3)	--	--
Control +	1944 ± 120	2877 ± 749	1197 ± 57	3605 ± 34	2033 ± 236	1700 ± 311	1836 ± 117	671 ± 25
MeOH Ext. Scapes	−S9 ^b^	+S9 ^d^	−S9 ^b^	+S9 ^d^	−S9 ^a^	+S9 ^d^	−S9 ^c^	+S9 ^e^
0	24 ± 2	31 ± 3	134 ± 2	235 ± 10	185 ± 11	105 ± 11	382 ± 30	248 ± 3
0.62	--	--	--	--	--	--	468 ± 10 (1.2)	370 ± 26 (1.5)
1.25	--	--	--	--	--	--	427 ± 33 (1.1)	320 ± 25 (1.3)
1.87	47 ± 2 (1.5)	32 ± 2 (1.5)	384 ± 65 (**2.3**)	837 ± 77 (2.6)	382 ± 21 (1.6)	180 ± 6.1 (1.7)	--	--
2.50	--	--	--	--	--	--	492 ± 138 1.3)	377 ± 13 (1.5)
3.75	41 ± 1 (1.2)	34 ± 6 (1.6)	235 ± 132 (1.4)	909 ± 180 (2.9)	187 ± 12 (0.9)	196 ± 10 (1.9)	493 ± 21 (1.3)	321 ± 61 (1.3)
7.50	61 ± 4 (1.9)	37 ± 4 (1.7)	172 ± 46 (1.0)	708 ± 73 (2.2)	151 ± 24 (0.7)	194 ± 7 (1.9)	--	--
11.25	41 ± 6 (1.3)	41 ± 2 (1.9)	30 ± 19 (0.2)	580 ± 89 (1.8)	21 ± 2 (0.1)	192 ± 28 (1.8)	--	--
Control +	1944 ± 120	2877 ± 749	1465 ± 57	2010 ± 536	2033 ± 236	3221 ± 117	1836 ± 117	671 ± 25

* *p* < 0.05 (ANOVA); ** *p* < 0.01, M ± SD = mean and standard deviation. MeOH Ext.: methanolic extract; 0 = Negative control: DMSO – 75 μL/plate. Control +: Positive control: ^a ^Sodium azide (2.5 μg /plate); ^b^ NPD (4-nitro-*O*-phenylenediamine – 10 μg/plate); ^c ^Mitomycin (0.5 μg /plate); ^d^ 2-Anthramine (1.25 μg /plate); ^e ^2-Aminofluorene (10 μg /plate). Values in brackets (MI) ≥ 2 indicate mutagenicity.

## 3. Experimental

### 3.1. Chemicals

HPLC-grade methanol was purchased from JT Baker (Baker Mallinckrodt, Phillipsburg, NJ, USA). HPLC-grade water was prepared with a Millipore (Bedford, MA, USA) Milli-Q purification system.

### 3.2. Plant Material

Capitulae and scapes of *P. chiquitensis* were collected in March 2010, in Serra do Cipó, Minas Gerais State, Brazil, geographical coordinates of 18°18'00.39"S, 43°41'06.46"W and authenticated by Professor Dr. Paulo Takeo Sano of the São Paulo University (USP), SP. A voucher specimen (3736 SPF) was deposited at the Herbarium of the IB-USP. 

### 3.3. Extraction

Dried and powdered capitulae (256 g) and scapes (176.7 g) of *P. chiquitensis* were separately extracted by maceration at room temperature with methanol. The solutions were evaporated to dryness under vacuum to give 13.2 g of crude methanol extract of capitulae (7.4%), and 15.7g of crude methanol extract of scapes (6.1%).

### 3.4. Sample Preparation

The methanol extracts of capitulae and scapes of *P. chiquitensis* were processed as reported in Santos *et al*. [13]. The crude extract (1 g) was dissolved in methanol (10 mL) and the mixture was centrifuged for 5 min at 3,200 rpm. The supernatant was filtered through a nylon membrane disk 22, 25 mm diameter, 0.22 μm pore size (Flow Supply, Cotia, SP, Brazil).

### 3.5. Isolation of Compounds and Characterization

The dried methanolic extract of capitulae (3.0 g) was dissolved in 15 mL MeOH and centrifuged for 10 min at 3500 rpm, twice. The combined supernatants were fractionated on a Sephadex LH-20 column (56 cm × 3 cm), using MeOH (1.5 L) as mobile phase, affording 302 fractions (7 mL) each.

The Sephadex fraction 24 (10 mg) afforded the pure compound 5 (10 mg, from the capitulae). Fractions 47–54 (161 mg) were separated by semi-preparative HPLC IR, on a Phenomenex Synergi Hydro RP 80 column (40 × 250 × 10.00 mm i.d., 4 μm),with injected volume 40 μL, at a flow rate 2.0 mL·min^−1^. The mobile phase consisted of 95% water (eluent A) and 5% methanol (eluent B), containing 0.1% acetic acid, in isocratic mode and afforded the compound **4a** (4 mg).

NMR analyses and 2D experiments of the compounds were run on a Varian® INOVA 500 operating at 500 MHz for ^1^H and 125 MHz for ^13^C (11.7 T), using TMS as internal standard.

### 3.6. Standard Solutions

Standard substances were obtained from a collection in our laboratory (**8**, **9**, **11**, **12**, **7a** and **9a** for naphthopyranones and **1**, **2** and **5** for flavonoids) isolated previously from Eriocaulaceae species and used as external standards. Analysis of these compounds by HPLC revealed a purity of 98.5% in the standards. These standards and the compounds isolated from the methanolic extract of the capitulae from *P. chiquitensis* were utilized as external standards in tests to identify the compounds in the methanolic extract of the scapes from *P. chiquitensis*.

### 3.7. HPLC-ESI-IT-MSn Analyses

The methanol extracts of *P. chiquitensis* (capitulae and scapes) were analyzed separately by in-line HPLC-ESI-IT-MS^n^, using a SURVEYOR MS micro system coupled in-line to an LCQ Fleet ion-trap mass spectrometer (Thermo Scientific). HPLC separation was conducted on a Phenomenex Luna RP 18 column (250 × 4.6 mm i.d. 5 micron) using a gradient mobile phase with a flow rate of 0.8 mL·min^−1^ of water (A) and methanol (B) plus 0.1% acetic acid. Initial conditions were 5% (B) increasing to reach 100% (B) and hold at 100% (B) at 80 min and held at 100% (B) for 10 min. 

The column effluent was split into two by means of an in-line T junction which sent it both to ESI-MS^n^ and UV-DAD; 80% was sent to the UV-DAD detector and 20% was analyzed by ESI-MS^n^ in negative ion mode with a Fleet LCQ Plus ion-trap instrument from Thermo Scientific. The capillary voltage was set at −20 kV, the spray voltage at −5 kV and the tube lens offset at 100 V, sheath gas (nitrogen) flow rate at 80 (arbitrary units) and auxiliary gas flow rate at 5 (arbitrary units). Data were acquired in MS1 and MSn scanning modes. The capillary temperature was 275 °C. Xcalibur 2.1 software (Thermo Scientific) was used for data analysis.

### 3.8. ESI-MS^n^ Analysis

Each isolated compound was subjected to negative ESI-MS^−1^ analysis under the same conditions as those used for HPLC-ESI-IT-MS^n^ analysis. Each compound was dissolved in methanol and infused in the ESI source by a syringe pump (flow rate 5mL/min). Nitrogen was used both as drying gas, at a flow rate of 60 (arbitrary units) and as nebulizing gas. Ion spray voltage was 5 kV and the tube lens offset was −55 V. The nebulizer temperature was set at 275 °C, and a potential of −4 V was used on the capillary. Negative ion mass spectra were recorded in the range *m/z* 150–2,000. The first event was a full-scan mass spectrum to acquire data on ions in the *m/z* range. The second event was an MS/MS experiment in which data-dependent scanning was carried out on deprotonated molecules of the compounds, at collision energy of 20% and activation time of 30 ms. 

### 3.9. Salmonella Mutagenicity Assay (Ames Test)

Chemicals: Dimethylsulfoxide (DMSO), nicotinamide adenine dinucleotide phosphate sodium salt (NADP), D-glucose-6-phospate disodium salt, L-histidine monohydrate, and D-biotin were purchased from Sigma Chemical Co. (St. Louis, MO, USA).

Standard Mutagens: Sodium azide, 2-anthramine, mitomycin and 4-nitro-*O*-phenylenediamine (NPD) were also obtained from Sigma. Oxoid Nutrient Broth N° 2 (Oxoid, UK) and Difco Bacto Agar (Difco, Oxoid, Basingstoke, HAM, UK) were used for the preparation of bacterial growth media. All other reagents used to prepare buffers and media were from Merck (Whitehouse Station, NJ, USA) and Sigma. 

### 3.10. Experimental Procedure

Test substances were first incubated for 20–30 min with the *S. typhimurium* strains TA100, TA98, TA97a and TA102, with or without metabolic activation by the addition of S9 mix [34]. *S. typhimurium* strains were kindly provided by Dr. B. Ames, University of California, Berkeley, CA, USA. The samples tested were the methanolic extracts of capitulae and scapes at four different doses in the range 0.60–11.25 mg/plate, dissolved in DMSO. The concentrations used were based on the bacterial toxicity established, in a preliminary test. The upper limit of the dose range tested for mutagenicity was either the highest non-toxic dose or the lowest toxic dose determined in the preliminary test. Toxicity was apparent either as a reduction in the number of His*+* revertants in the Ames test or as an alteration in the auxotrophic background lawn. The various doses tested were added to 500 μL of buffer (pH 7.4) and 100 μL of bacterial culture and then incubated at 37 °C for 20−30 min. Next, 2 mL of top agar was added to the mixture and the whole poured on to a plate containing minimal agar. The plates were incubated at 37 °C for 48 h and the His+ revertant colonies were counted manually. The influence of metabolic activation was tested by adding 500 μL of S9 mixture (4%) to the bacterial culture in place of the buffer. The S9-mix was freshly prepared before each test with an Aroclor-1254-induced rat liver fraction purchased (lyophilized) from Moltox (Molecular Toxicology Inc., Boone, NC, USA). All experiments were analyzed in triplicate. The standard mutagens used as positive controls in experiments without S9-mix were 4-nitro-*O*-phenylenediamine (10 μg/plate) for TA98 and TA97a, sodium azide (1.25 μg/plate) for TA100 and mitomycin (0.5 μg/plate) for TA102. In tests with metabolic activation, 2-anthramine (0.125 μg/plate) was used for TA98, TA100 and TA97a and 2-aminofluorene (10 μg /plate) for TA102. DMSO (75 μL/plate) served as the negative (solvent) control. The statistical analysis was performed with the Salanal computer program, adopting the Bernstein model [35]. The mutagenic index (MI) was also calculated for each dose, as the average number of revertants per plate divided by the average number of revertants per plate of the negative (solvent) control. A sample was considered mutagenic when MI ≥ 2 for at least one of the tested doses and the response was dose dependent [5,20,21].

## 4. Conclusions

This paper described a sensitive, specific and simple method for characterization of major constituents of *P. chiquitensis* extracts. Eighteen flavonoids of the types flavanonol and flavonol and six naphthopyranones were identified or tentatively characterized in one LC-MS^n^ run. Results obtained by this method could significantly decrease the time required for the identification of some known flavonoids present in *P. chiquitensis* extracts; furthermore, isolation and purification of authentic reference were unnecessary. This methodology also provides chemical support for the chromatographic *fingerprint* technology and could facilitate the taxonomic study of the genus *Paepalanthus. *It is also suggested that the concentration of flavonoids and naphthopyranones found in the capitulae and scapes of *P. chiquitensis* can explain the mutagenic activity towards strain TA97a.
